# The effect of iliotibial band surgery at the hip: a systematic review

**DOI:** 10.1186/s12891-023-06169-4

**Published:** 2023-01-28

**Authors:** Simon Storgaard Jensen, Kristina Lund, Jeppe Lange

**Affiliations:** 1 Department of Orthopaedic Surgery, Regionshospitalet Gødstrup, Central Denmark Region, Herning, 7400 Denmark; 2 Aarhus University, Aarhus, 8000 Denmark; 3 Institut for Klinisk Medicin, Aarhus University, Aarhus, 8000 Denmark; 4 Department of Orthopaedic Surgery, Regionshospitalet Horsens, Central Denmark Region, Horsens, 8700 Denmark

**Keywords:** Adult, Hip Joint/surgery, Fascia Lata/surgery, Bursitis/surgery, Pain/surgery

## Abstract

**Background:**

Current literature presents a variety of surgical interventions aimed at modifying the iliotibial band (ITB) at the hip to relieve lateral hip pain (LHP). However, a focus towards the hip abductors as a main driver in LHP has evolved in the last decade, which could influence the indications for isolated ITB surgery. No previous review has been undertaken to evaluate isolated ITB surgery in LHP cases.

**Purpose:**

The purpose of this systematic review was to evaluate isolated ITB surgery in LHP patients in relation to pain, snapping, use of non-surgical treatments postoperatively, and repeated surgery.

**Methods:**

The study was reported in accordance with Preferred Reporting Items for Systematic Reviews and Meta-Analyses. The study was registered in Prospero (CRD42021216707) prior to initiation. A systematic search of literature on PubMed and Embase as well as bibliography screening on adult patients undergoing isolated ITB surgery with or without additional bursectomies was performed. Due to the lack of reliable data, no meta-analysis was performed.

**Results:**

A total of 21 studies (360 patients) were considered eligible for inclusion. The snapping and non-snapping group consisted of 150 and 210 patients, respectively. The mean follow-up time in the snapping group was 30 months and 19 months in the non-snapping group. Utilizing different surgical techniques, complete pain relief was not achieved in 12% of patients in the snapping group and 36% of the patients in the non-snapping group. In the snapping group, snapping was eliminated in 95% of patients, and five of 150 patients (3%) had repeated surgery. Eight of nine non-snapping studies reported information regarding repeated surgery, in which seven of 205 patients (3%) received repeated surgery.

**Conclusion:**

ITB surgery at the hip remains widely adopted, although only level 4 studies are available, and little information exists on the long-term clinical, as well as patient reported outcomes. Based on the available data, we found indication of a positive short-term outcome in LHP with snapping regarding elimination of snapping, pain reduction, reuse of non-surgical treatment, and repeated surgery. In LHP with no snapping, we found limited evidence supporting ITB surgery based on current literature.

**Supplementary Information:**

The online version contains supplementary material available at 10.1186/s12891-023-06169-4.

## Introduction

Lateral Hip Pain (LHP) is caused by numerous extra-articular hip pathologies centered around the greater trochanter [48, 58]. Current literature indicates that LHP is a common complaint, with a prevalence reported near 6 per 1000 adults [35, 64]. LHP negatively affects patients at the level of end-stage hip osteoartrosis [48].

LHP covers a variety of underlying pathologies, not always with clear diagnostic parameters, and are historically attributed mainly to greater trochanteric bursitis (GTB) and coxa saltans externa (CSE), and more recently hip abductor tendon pathology [50, 51, 67]. CSE is found as frequently as 10% in the young adult population [30, 47]. Most often among athletes, females, and young adults [6, 25, 44].

The iliotibial band (ITB) is a tendinous band of muscle insertions from the gluteus maximus and the tensor fascia lata, which surpasses the greater trochanter. The ITB may be thickened in this distinct area [36], and is often directly related to GTB and CSE. Recent Magnetic Resonance Imaging studies indicate that a thickening of the ITB at the level of the greater trochanter can occur due to repetitive sliding [24, 67]. This can contribute to a snapping sensation when the hip is flexed and then extended and/or rotated. Furthermore, the mechanical irritation caused by a tight and thickened ITB is believed to induce inflammation in the greater trochanteric bursa, located between the ITB and the greater trochanter, and this relates to pain found in GTB [1, 30, 53, 56, 67]. However, pain and dysfunction do not always correlate with clinical findings [49], as some of the CSE patients describe the snapping without any reported discomfort or accompanying pain [30, 56, 65].

Many treatments of LHP, both surgical and non-surgical, have been aimed at the ITB, as it surpasses the greater trochanter. Non-surgical treatment is usually applied as first line treatment and includes rest, physiotherapy, stretching of the ITB, anti-inflammatory medication and corticosteroid injections into the trochanteric bursa, which is repeated if necessary [7, 15, 16, 19, 31, 33, 46, 67]. However, in cases refractory to non-surgical treatment, patients are often referred to ITB surgery [58, 67].

Current literature presents a variety of surgical interventions aimed at modifying the ITB to relieve the pain and/or snapping in LHP, the majority based on the original Z-plasty approach presented by Brignall and Stainsby in 1991 [7]. The ITB-techniques are uniformly believed to ensure less friction and inflammation around the greater trochanter, thereby reducing pain and snapping. These are performed open as well as endoscopic, with or without additional bursectomy.

The purpose of this systematic review was to evaluate iliotibial band surgery at the hip in LHP patients in relation to i) reduction of pain, ii) elimination of snapping, iii) use of non-surgical treatments beyond six months postoperatively, iv) repeated surgery.

The objectives of this systematic review were: 1) LHP patients without a clear description of snapping would not benefit from ITB surgery, 2) LHP patients with snapping would benefit from ITB surgery.

## Methods and materials

The study was reported in accordance with Preferred Reporting Items for Systematic Reviews and Meta-Analysis [32, 37]. The study was registered in Prospero (CRD42021216707) prior to initiation.

### Eligibility criteria

Patients eligible for inclusion in the review were i) adult patients (≥ 18 years); ii) diagnosis of CSE, GTB, LHP or greater trochanteric pain syndrome (GTPS) as defined in the individual study; iii) undergone isolated open or endoscopic ITB surgery, with or without additional bursectomy due to ii); iv) minimum follow-up period of six months after iii); iv) no previous hip surgery performed.

Inclusion and exclusion criteria are described in Table 1.

**Table 1:** Inclusion and exclusion criteria

**Inclusion criteria**	**Exclusion criteria**
Adult population	Reviews
Follow-up of at least six months	< 18 years
Iliotibial band surgery as intervention	Non-surgical intervention
Refractory to conservative treatment	Other languages than Danish and English
Unilateral or bilateral LHP w/ or w/o snapping	Hip fractures or hip arthroplasty or THA
	Previous hip surgery
	Internal snapping hip, intraarticular hip disease or gluteus tendon pathology
	Case series < 5 patients
	Unable to receive patient information from authors

The inclusion and exclusion criteria applied as key concepts in Covidence

The table is based on PROSPERO registration

### Information sources

**Fig. 1:**
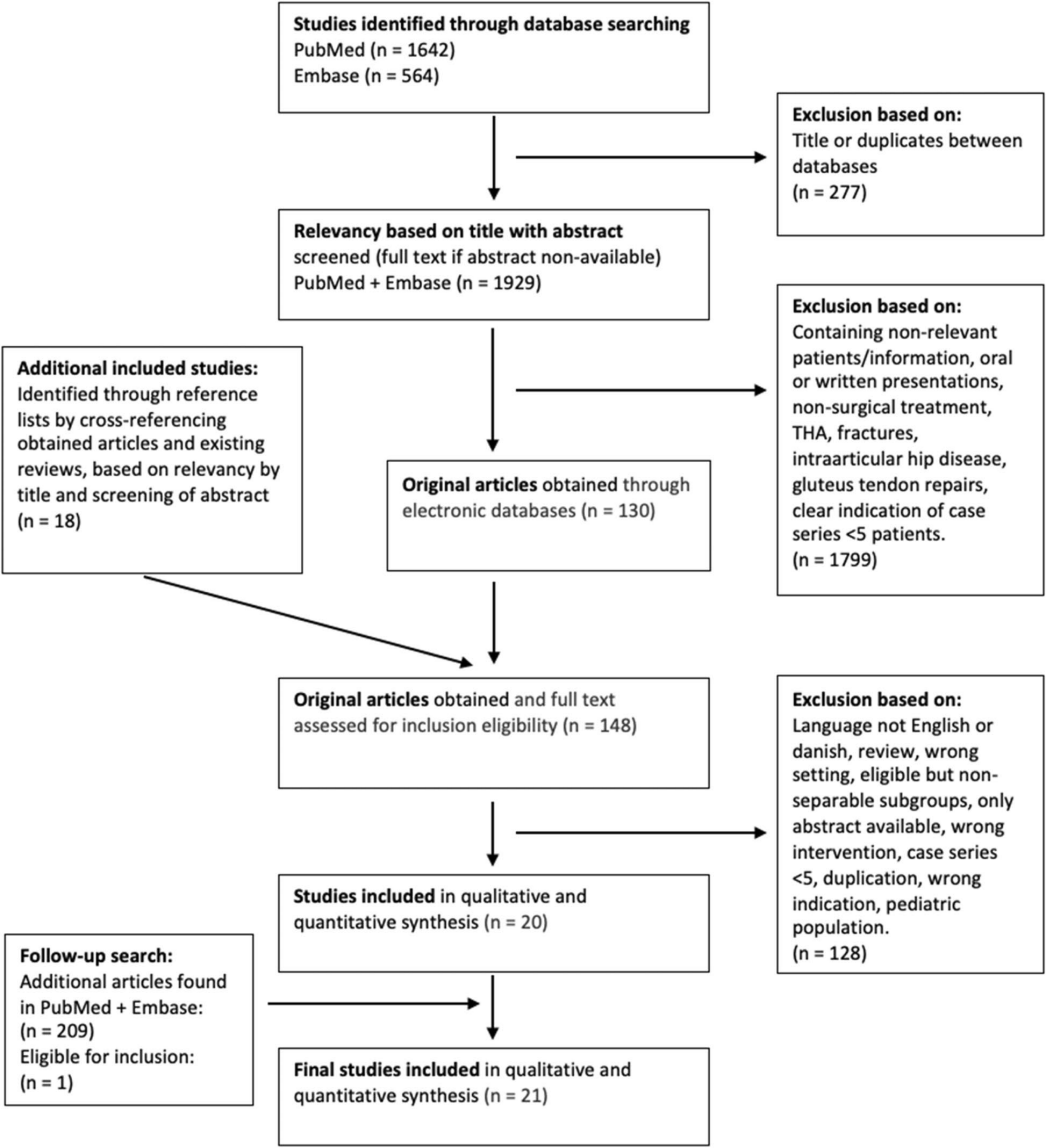
PRISMA Flow Diagram. Abbreviation: PRISMA, Preferred Reporting Items for Systematic Reviews and Meta-Analysis

Studies were identified by electronic database searching of PubMed (1954-) and Embase (1971-). Main search was performed in December 2020. To ensure up-to-date results, a follow-up search was conducted in October 2021 (Fig. 1).

The reference lists of included studies and identified relevant reviews [20, 21, 26, 38, 47, 48, 50, 56, 58, 67] were assessed for potentially relevant studies, not identified in the database search (“snowballing”).

### Search strategy

The search strategy was developed by all the authors in collaboration with a university research librarian (Table 2).

**Table 2:** Search strategy

Search preformed in the following numerical order (PubMed/Embase)	
#1	Snapping hip OR snapping hip syndrome
#2	External snapping hip
#3	Coxa saltans OR coxa saltans externa
#4	Greater trochanteric pain
#5	Lateral hip pain
#6	Iliotibial band syndrome
#7	Surgery OR surgical correction
#8	Endoscopic surgery OR open surgery
#9	Z-plasty OR z plasty
#10	Iliotibial release OR iliotibial band release OR iliotibial band lengthening
#11	Surgical release
#12	#1 OR #2 OR #3 OR #4 OR #5 OR #6
#13	#7 OR #8 OR #9 OR #10 OR #11
#14	#12 AND #13

The search strategy as presented was applied as key concepts. No limits applied

The search string was based on two components. One component regarding the condition of the hip, and one component regarding the surgical intervention. When building the search string, we could not identify relevant medical subject headings for the two components. A pragmatic approach was taken in the first component, and the terms used were those defined in known publications, such as LHP, GTPS, GTB, CSE.

The surgical intervention component also varies throughout the literature, e.g., Z-plasty, N-plasty, Diamond-shaped release, all consisting of modified ITB release techniques. To ensure a wide inclusion, a broader approach to terms included were used, e.g. ‘surgery’, ‘surgical release’ and ‘ITB release’.

### Selection of studies and data collection process

All study designs were accepted for inclusion in this review. Only patient data reported in full text articles were included for analysis. Only English-language publications were evaluated. Case series of less than five patients were excluded. Selected patients from studies with eligible subgroups were included, if the relevant data on selected participants could be obtained. We contacted authors in individual studies to provide specific patient data on a study level if needed.

Covidence [18, 60] was used as a stepwise tool to evaluate and manage studies from the database searches. Each assessment step in Covidence was done blinded between two authors. Following full assessment in each step, the two authors compared results. Any disagreement was resolved by consensus by all three authors. All studies were individually assessed for overlapping patient data before final inclusion.

All studies were assessed by their title through the electronic database search by two of the authors. If deemed relevant, the abstract was retrieved. If the abstract indicated eligibility, the full text was obtained. In cases where no abstract was available, and the title indicated eligibility, the full text was obtained and assessed. If full text was not available, the study was excluded.

Following the evaluating of the studies in Covidence, the data extraction was conducted using Microsoft Excel spreadsheet. First, the data was extracted from the individual studies by the authors independently and blinded to each other’s extraction. Secondly, all data was entered into an Excel spreadsheet for tabulation and data management by consensus.

We had a priori defined two distinct LHP groups for stratification according to the defined objective.

### Data items

The following variables were extracted from included publications: 1) main intervention – type of ITB surgery (additional bursectomy, open or endoscopic); 2) study outcomes – absence of conservative treatment, reduction of pain, elimination of snapping, and absence of repeated surgery; 3) study demographics – first author, publication year, defined in- and exclusion criteria, study design and data collection perspective; 4) study population demographics – surgical measurement, previous use of conservative treatment and type, study size, age, gender, primary indication and symptoms duration; 5) perioperative setting – follow-up period and patient related outcome measures (PROM) and pain scores.

If the number of hips affected was not specified in the studies, the condition was assumed unilateral.

### Quality and bias assessment

We had planned to apply the Quality Assessment Tool for Quantitive Studies by Effective Public Health Practice Project for a study-by-study assessment. However, prior to evaluating the methodological quality of the studies, we applied the GRADE tool to evaluate the quality of evidence. All identified studies in this review were evaluated as *very low* GRADE level of evidence [4], with a very low degree of clinical practice recommendation and where the true effect is probably markedly different. Therefore, no formal quality assessment of each individual study was undertaken nor reported.

A narrative bias assessment of the methodological and clinical limitations for the included studies was performed with a focus on key study features; 1) patient cohort – in- and exclusion criteria; 2) follow-up – adequate defined as six months or more; 3) outcome – sufficient use of validated scores for outcome [34]; and 4) intervention – clear description of surgical intervention and technique [47].

### Effect measures

The extracted outcomes were 1) pain reduction; 2) elimination of snapping; 3) repeated use of conservative treatment; 4) repeated ITB surgery.

The elimination of snapping, repeated use of conservative treatment, and the need of a repeated surgery was pragmatically evaluated as yes or no, with no differentiation of the concrete type of evaluation or intervention.

### Synthesis of data

As the level of evidence in the identified literature was very low, no formal synthesis of data (meta-analysis) was performed. Descriptive statistics were used to present the study characteristics, as well as surgical intervention and outcome.

## Results

### Study selection

Overall, 21 studies with a total of 360 patients were considered eligible for inclusion (Fig. 1). Of the 21 studies, 18 were identified by the electronic-database searches, and three were identified by “snowballing”. Specifically, we excluded studies [2, 23, 61, 66] which, after full text review, did not fulfill our inclusion criteria. We also excluded potential eligible studies [7, 9, 17, 29, 45, 54, 55, 69] in which individuals with age < 18 years, or previous hip surgery, could not be separated in the study population after attempted contact with the authors.

### Study characteristics

All studies reported surgical interventions with isolated ITB-releasing techniques at the hip. No studies compared surgical intervention with a nonsurgical treated control group or with another ITB surgery.

One study was a randomized controlled trial (RCT) comparing ITB surgery with or without radiofrequency microdebridement [3], but all included patients received identical ITB surgery, why the randomization was not deemed relevant to this review, and the study was included as a cohort. One prospective study [14] with information regarding study aims and measures prior to patient inclusion was found. The remaining 19 studies were retrospective case-series, some as defined at a study level, while some studies did not provide clearly detailed information regarding data collection. Of all 21 studies, the snapping group averaged 13 patients per study (range 5–48), and the non-snapping averaged 23 patients per study (range 5–58).

Four studies [3, 12–14] provided detailed information on inclusion and exclusion criteria. Six studies [24, 40, 44, 59, 68, 70] specified some degree of information, like previous hip pathologies or surgery.

The remaining 11 studies provided only very limited to no information on in- and exclusion criteria.

Two studies provided information on comorbidities (medical history and smoking) [3, 14], while three studies [11, 40, 43] had some information on relevant patient characteristics (BMI and profession). The remaining 16 studies did not offer specific details on demographics and comorbidities.

Duration of symptoms differed among the studies. Information regarding duration of symptoms was stated in 15 of 21 studies ranging from two months to “decades” of symptoms [62]. In the snapping group, 11 of 12 studies stated the duration. In the non-snapping group, four of nine studies had a description of the duration. Preoperative use of conservative treatment was reported in 20 studies, and 15 of these stated the duration to be at least three months. It is not stated whether the 48 patients in Dai et al [11] received conservative treatment. All remaining patients apart from one individual in Thomassen et al [59] received conservative treatment before ITB surgery. The applied type of conservative treatment differed among the studies, but it was consistent with generally accepted standards [48]. Some merely used steroid-injections [10, 62] while a few stated the use of conservative treatment without defining it [39, 68].

Overall, six studies originated from Europe (*n *= 122) [12, 14, 43, 52, 59, 70], six from North America (*n* = 56) [8, 22, 44, 57, 62, 71], five from Asia (*n* = 87) [11, 24, 39, 40, 68], three from Australia/New Zealand (*n* = 97) [3, 10, 13] and one from South America (*n* = 8) [42]. The studies were published between 1986–2020 (snapping group) and 1979–2021 (non-snapping group). No tendency regarding uneven distribution of publication year was found.

Although not quantified in this systematic review, the risk of both information and selection bias was believed to be very high due to the majority of included studies being retrospective case-series with small sample-sizes. Also, the risk of publication bias with lack of publications with poor surgical outcomes is high, although not graphically evaluated via funnel plot. We did not believe it of value to the conclusions in this systematic review to quantify the above further.

### Results of individual studies

The *snapping* group consisted of patients with a clear description of snapping in each individual case in the study. The *non-snapping* group consisted of patients without a clear description of snapping in individual cases.

#### Snapping group

The snapping group comprised 150 patients from 12 studies (Open surgery, *n* = 55 patients, Endoscopic surgery, *n* = 95). Seven studies described additional bursectomy. The study characteristics are presented in Table 3, and the surgical outcome characteristics are summarized in Table 4.

**Table 3:** Study characteristics of the snapping group

**Year & Author**	**Study design**	**Syndrome** **(Study description)**	**Inclusion duration** **Year, months**	**Patients included** **(male)**	**Mean age** **(range)**	**Duration of symptoms, m (range)**	**Duration of conservative treatment, m**	**Mean** **follow-up, m** **(range)**
1986, Zoltan et al [71]	Retrospective	CSE + GTB	n/a	7 (4)	25 (21–33)	(4–48)	n/a	55 (12–76)
2004, Provencher et al [44]	Retrospective	CSE	4y, 8 m	8 (4)	25.6 (21–38)	25.2 (16–39)	13	22,9 (7–38)
2004, White et al [62]	Retrospective	CSE	7y, 8 m	11 (3)	41.1 (21–65)	(2 m-decades)	n/a	32.5 ^A^ (9–74)
2006, Ilizaliturri et al [22]	Retrospective	CSE	2y, 4 m	10 (1)	26 (21–35)	31 (10–38)	n/a	24 (12–36)
2011, Nam et al [39]	Retrospective	CSE	6y, 4 m	7 (5)	26 (21–33)	192 (120–360)	n/a	87 (47–122)
2012, Sayed-Noor et al [52]	Retrospective	CSE	2y, 2 m	5 (2)	32.8 (20–44)	n/a	6–12	12 (12–12)
2013, Polesello et al [42]	Retrospective	CSE	2y, 0 m	8 (1)	35 (18–55)	36 (16–84)	> 3	32 (22–45)
2013, Zini et al [70]	Retrospective	CSE	6y, 3 m	14 (3)	25.7 (18–37)	18,14 (10–48)	> 6	39.5 (12–84)
2014, Yoon et al [68]	Retrospective	CSE	1y, 10 m	7 (2)	35 (25–49)	36 (24–120)	> 3	19 (12–33)
2017, Park et al [40]	Retrospective	CSE + GTB	2y, 8 m	17 (17)	20.8 (20–22)	28.5 (2,8–120)	> 3–4	18.2 (8–24)
2018, Dai et al [11]	Retrospective	CSE + GT	6y, 1 m	48 (18)	20.8 (18–28)	162 (96–228)	n/a	28.3 (24–48)
2020, Kim et al [24]	Retrospective	CSE	1y, 7 m	8 (8)	23.6 (18–41)	10.75 (4–24)	> 6	> 6 (n/a)

*Abbreviations: n/a* Not available, *m* Months, *CSE* Coxa saltans exterma, *GTB* Greater trochanteric bursitis, *GT* Gluteal tendinopathy, *ITB* Iliotibial band

**A-**Follow-up was based on 16 patients not excluding the 5 patients < 18 years old

**Table 4:** Surgical outcome characteristics of the snapping group

**Year, author**	**Syndrome**	**Surgical** **intervention (study description)**	**Additional** **bursectomy**	**Surgical** **approach**	**Patients included hips**	**PROM, preoperative, mean (range)**	**PROM, postoperative, mean (range)**	**Pain score, preoperative**	**Pain score, postoperative**	**Relief of pain; complete, partial, none (patients)**	**Snapping eliminated**	**Complications** **(yes, no)**	**Repeated surgery (n)**	**Reuse of conservative treatment (n)**
1986, Zoltan et al [71]	CSE + GTB	Ellipsoid-shaped release	Yes	Open	7, 7	n/a	n/a	n/a	n/a	1,2,2	4/5	1, 6	1	n/a
2004, Provencher et al [44]	CSE	Proximal Z-plasty	n/a	Open	8, 9	Ober: 9/9 hips	Ober: 0/9 hips	n/a	n/a	8,0,1^A^	9/9^A^	1, 7	1	n/a
2004, White et al [62]	CSE	ITB-release transverse step cuts	Yes	Open	11, 12	n/a	n/a	n/a	n/a	8,0,3	9/10	2, 8	2	n/a
2006, Ilizaliturri et al [22]	CSE	Diamond-shaped-release	Yes	Endoscopic	10, 11	WOMAC = 78 (78–87)	WOMAC = 94 (89–96)	n/a	n/a	10,0,0	10/11^A^	0, 10	0	n/a
2011, Nam et al [39]	CSE	Modified z-plasty	n/a	Open	7, 14	n/a	n/a	n/a	n/a	7,0,0	14/14^A^	0, 7	0	n/a
2012, Sayed-Noor et al [52]	CSE	Distal Z-plasty	n/a	Open	5, 5	n/a	n/a	n/a	n/a	5,0,0	5/5	1, 4	0	n/a
2013, Polesello et al [42]	CSE	GMT-release	n/a	Endoscopic	8, 9	mHHS = 61.3 (45–70)	mHHS = 77.6 (62–93)	n/a	n/a	7,1,1^A^	7/9^A^	1, 7	1	n/a
2013, Zini et al [70]	CSE	ITB-release + GMT-release	Yes	Endoscopic	14, 14	HHS = n/a	HHS = 97.8 (94–100)	VAS = 5.57 (5–7)	VAS = 0.57 (0–2)	8,6,0	14/14	0, 14	0	n/a
2014, Yoon et al [68]	CSE	Diamond-shaped-release + gluteal sling release	Yes	Endoscopic	7, 10	mHHS = 68.2 (43–73)	mHHS = 94.8 (89–100)	VAS = 6.8 (6–9)	VAS = 0.2 (0–2)	6,1,0	10/10^A^	0, 7	0	n/a
2017, Park et al [40]	CSE + GTB	N-plasty	Yes	Open	17, 24	mHHS = 69.5 (44–82.5)	mHHS = 97.8 (92,4–100)	VAS = 6.77 (6–9)	VAS = 0.1 (0,2)	17,0,0	24/24^A^	1, 16	0	n/a
2018, Dai et al [11]	CSE + GT	ITB-release + GMC-release	n/a	Endoscopic	48, 96	HHS = 81.5 (SD = 7.2)	HHS = 99.9 (SD = 0,7)	VAS = n/a	VAS = 2.8 (SD = 0.7)	48,0,0	92/96^A^	1, 47	0	n/a
2020, Kim et al [24]	CSE	Diamond-shaped-release	Yes	Endoscopic	8, 8	mHHS = 75.3 (66–84)	mHHS = 85 (77–91)	VAS = 4.3 (2–6)	VAS = 1,1 (0–2)	7,1,0	7/8	0, 8	0	n/a

*Abbreviations: n/a* Not available, *CSE* Coxa saltans exterma, *GTB* Greater trochanteric bursitis, *GT* Gluteal tendinopathy, *ITB* Iliotibial band, *GMT* Gluteus maximus tendon, *GMC* Gluteal muscle contracture, *PROM* Patient related outcome measure, *HHS* Harris hip score, *mHHS* Modified harris hip score, *VAS* Visual analog scale, *WOMAC* The Western Ontario and McMaster Universities Osteoarthritis Index

**A** Results provided in number of hips and not number of patients

#### Non-snapping group

The non-snapping group comprised 210 patients from nine studies (Open surgery, *n* = 36 patients, Endoscopic surgery, *n* = 174). Additional bursectomy was described in eight studies. The study characteristics are presented in Table 5, and the surgical outcome characteristics are summarized in Table 6.

**Table 5:** Study characteristics of the non-snapping group

Year & Author	Study design	Syndrome (study description)	Inclusion duration	Patients included (male)	Mean age (range)	Duration of symptoms, m (range)	Duration of conservative treatment, m	Mean follow-up, m (range)
**1979,** **Brooker et al **[8]	Retrospective	GTB	n/a	5 (5)	n/a (50–65)	n/a	24–60	12 (12–12)
**1997,** **Slawski et al **[57]	Retrospective	GTB	n/a	5 (1)	40.3 (24–54)	45.6 (24–84)	> 12	20 (12–30)
**2007,** **Craig et al **[10]	Retrospective	GTPS (GTB)	n/a	15 (1)	60 (36–73)	56.4 (7–180)	n/a	47 (5–69)
**2009,** **Pretell et al **[43]	Retrospective	GTB	6y, 5 m	11 (1)	54.6 (32–74)	22 (12–60)	> 12	43 (15–84)
**2014, Domínguez et al **[12]	Retrospective	GTPS	2y, 0 m	23 (4)	51.3 (SD: 13.3)	n/a (6–24)	> 3	12 (12–12)
**2016,** **Drummond et al **[13]	Retrospective	GTPS	3y, 3 m	49 (7)	65 (27–89)	n/a	> 6	20,7 (5–41)
**2019, Thomassen et al **[59]	Retrospective	GTPS	2y, 10 m	11 (5)^B^	57 (43–71)	n/a	> 12	28 (15–42)
**2020, Blakey et al **[3]	Prospective^C^	GTPS (GT)	3y, 9 m	33 (3)	57.7 (SD: 8,3)	n/a	> 6	12 (12–12)
**2021,** **Dzidzishvili et al **[14]	Prospective	GTPS (GTB)	1y, 0 m	58 (7)	56.9 (28–80)	n/a	> 6	12 (12–12)

*Abbreviations: n/a* Not available, *m* Months, *y* Years, *GTPS* Greater trochanteric pain syndrome, *GTB* Greater trochanteric bursitis, *GT* Gluteal tendinopathy, I*TB* Iliotibial band

**A** 20 patients were not available for follow up within 6 months and were categorized excluded rather than lost to follow up

**B** One patients refused conservative treatment but was still included in the study

**C** RCT-study, but for the purpose of this review a prospective study

**Table 6:** Surgical outcome characteristics of the non-snapping group

**Year, author**	**Syndrome, snapping**	**Surgical** **intervention** **(Study description)**	**Additional** **bursectomy**	**Surgical** **approach**	**Patients included, hips**	**PROM, preoperative, mean (range)**	**PROM, postoperative mean (range)**	**Pain score, preoperative**	**Pain score, postoperative**	**Relief of pain; complete, partial, none**	**Snapping eliminated**	**Complications** **(yes, no)**	**Repeated surgery (n)**	**Reuse of conservative treatment (n)**
**1979,** **Brooker et al **[8]	GTB, n/a	ITB-release (T-shape)	n/a	Open	5, 5	HHS = 46 (n/a)	HHS = 88 (n/a)	n/a	n/a	4,1,0	n/a	n/a	n/a	n/a
**1997,** **Slawski et al **[57]	GTB, no	ITB-release (vertical)	Yes	Open	5, 7	HHS = 51.7 (45–56)	HHS = 95 (84–100)	n/a	n/a	1,2,1	IRLVT	0, 4	0	1
**2007,** **Craig et al **[10]	GTPS (GTB), n/a	Z-plasty	Yes	Open	15, 17	HHS = 46 (n/a)	HHS = 82 (n/a)	Pain-score = 11,7 ^A^	Pain score = 31.2 ^A^	8,8,1^B^	n/a	2, 13	1	n/a
**2009,** **Pretell et al **[43]	GTB, no	Distal Z-plasty	n/a	Open	11, 13	HHS = 61 (48–77)	HHS = 91 (76–95)	VAS = 8.3 (6–9.9)	VAS = 1.3 (0–7)	12,0,1^B^	IRLVT	2, 9	1	n/a
**2014, Domínguez et al **[12]	GTPS, yes	Diamond-shaped release	Yes	Endoscopic	23, 23	mHHS = 40.2 (n/a) WOMAC 63.3	mHHS = 86.3 (n/a) WOMAC 5.2	VAS = 8.1 (n/a)	VAS = 0.5 (n/a)	23,0,0	22/23	1, 22	0	n/a
**2016,** **Drummond et al**[13]	GTPS, n/a	ITB-release (vertical)	Yes	Endoscopic	49, 57	iHOT-33 = 23.8 (n/a)	iHOT-33 = 70.2 (n/a)	VAS = 7.8 (n/a)	VAS = 2.8 (n/a)	14,36,7^B^	n/a	0, 49	0	14^B^
**2019, Thomassen et al **[59]	GTPS, no	ITB-release (star-shaped)	Yes	Endoscopic	11^c^, 11	HHS = n/a	HHS = 73.8 (41–86)	NRS = 8 (7–9)	NRS = 4 (2–6)	0,11,0	IRLVT	1, 10	1	10
**2020, Blakey et al **[3]	GTPS (GT), n/a	Diamond-shaped release	Yes	Endoscopic	33, 33	mHHS = 58.2 (SD = 11.5)	mHHS = 78.8 (SD = 18.6)	VAS = n/a	VAS = 33.3-improvement	23,0,10	n/a	2, 31	2	n/a
**2021,** **Dzidzishvili et al **[14]	GTPS (GTB), n/a	ITB-Release (cross- shaped)	Yes	Endoscopic	58, 60	HOS = 24.1 (n/a)	HOS = 70.6 (n/a)	VAS = 9.6 (n/a)	VAS = 2.1 (n/a)	50,0,8	n/a	6, 52	2	6

*Abbreviations: n/a* Not available, *GTPS* Greater trochanteric pain syndrome, *GTB* Greater trochanteric bursitis, *ITB* Iliotibial band, *PROM* Patient related outcome measure, *IRLVT* Irrelevant, *HHS* Harris hip score, *mHHS* Modified harris hip score, *VAS* Visual analog scale, *NRS* Numeric rating scale, iHOT-33 International hip outcome tool, *HOS* Hip outcome score

**A** The study used a “pain score” ranging from 0–44

**B** Results provided in number of hips and not number of patients

**C** One patients refused conservative treatment but was still included in the study

In the snapping group, 68 of 150 (45%) patients were male, compared to 34 of 210 (16%) patients in the non-snapping group (*P* < *0,0001*). The average age in the snapping group was 26 years (95% CI: 25–28), compared to 58 years (95% CI: 57–59) in the non-snapping group (*P* < *0,0001*).

Mean follow-up time for all studies was 24 months (range 6—87 months), while the mean in the snapping group was 30 months (95% CI: 27–33), and the non-snapping group was 19 months (95% CI: 18–21).

#### Study outcomes

In relation to i) reduction of pain, five of 12 studies in the snapping group used visual analogue scale (VAS) [11, 24, 40, 68, 70], one used modified Harris hip score [42] and one used the Western Ontario and McMaster Universities Osteoarthritis Index [22]. The remaining five snapping group studies simply asked the patients if the pain had subsided [39, 44, 52, 62, 71]. The preoperative VAS-score of the five snapping group studies varied from 4 to 7, and the postoperative VAS-score varied from 0.1 to 3.

In the non-snapping group, six of nine studies used numeric rating scale (NRS) [59] or VAS [3, 12–14, 43], while one study used a not-validated “pain-score” [10], and two studies used Harris hip score [8, 57]. The preoperative VAS/NRS score of the six non-snapping group studies varied from 8 to 10 and the postoperative score varied from 0.5 to 4. We chose not to perform a compiled assessment across studies on pain due to the diverse nature of the reporting. Some studies reported pain relief per patient, and some per number of hips. Polesello et al [42] as example, included eight patients representing nine hips, with complete pain relief in seven hips, partial relief in one hip, and no relief in one hip. We chose to evaluate the two hips that did not have complete pain relief as representing one patient in this review. Similar evaluation was made regarding any study that reported in hips instead of patients [10, 13, 42–44]. Applying this approach, all calculations were made based on pain relief per patient, and within the individual study reported follow-up period, complete pain relief was not achieved in 12% of patients in the snapping group and 36% of the patients in the non-snapping group.

In relation to ii) elimination of snapping, 143 of 150 patients (95%) in the snapping group reported resolution of this.

In relation to iii) use of non-surgical treatments beyond six months postoperatively, repeated use of non-surgical treatment was not directly reported in any of the studies in the snapping group. A total of 24 of 123 (20%) patients in four studies in the non-snapping group had repeated use of conservative treatment [13, 14, 57, 59], whereas five studies did not report on reuse of conservative treatment (*n* = 87) [3, 8, 10, 12, 43].

In relation to iv) repeated surgery, repeated surgery was reported in five of 150 patients (3%) in the snapping group. In the non-snapping group, eight of nine studies reported information regarding repeated surgery, in which seven of 205 patients (3%) received repeated surgery. Brooker et al [8] did not report any information regarding repeated surgery.

## Discussion

The purpose of this systematic review was to evaluate adult patients with a surgical ITB-intervention at the hip to assess the value in LHP patients.

We have stringently evaluated all relevant and available literature on the topic, but we were only able to identify a limited number of studies on the topic, mainly smaller retrospective case-series, and as such very limited inference can be made from the included studies and their data. As isolated ITB surgery is widely adapted, and we believe the a priori defined outcomes of high clinical relevance, this is of concern.

Some observations can be made from the data extracted in this review.

First, we identified a difference in age and gender in patients with ITB surgery based on the group stratification, in that the snapping group were younger and had a higher frequency of males, whereas the non-snapping group consisted mainly of middle-aged women. This is in line with the clinical experience of the senior author [5].

In the snapping group, snapping was eliminated in 95% of patients, and the indication for ITB surgery to relieve snapping is accomplished in a vast majority of a highly selected group of patients. However, to conclude on the long-term effect of ITB surgery, a sufficient follow-up-period is required [20, 50]. Since most studies were small case series with a short follow-up (mean: 24 months), the results regarding long-term clinical and patient perceived outcome remain uncertain.

Robust and repeated reports applying validated pain outcome measures following ITB surgery compared to other interventions in LHP patients is not readily available in current literature, and as such no decisive interpretation can be made either for or against the use of pain reduction as an indication for ITB surgery. In our opinion, based on the data in this review, concerns for ITB surgery should arise in patients who report no snapping in addition to their LHP. We found that complete pain reduction was not achieved in 36% of the patients in the non-snapping group, despite a short, mean follow-up of only 19 months. To abstain from isolated ITB surgery in non-snapping cases is further justified by the current increasing clinical acceptance of hip abductor tendon pathologies as the true cause of pain in the majority of LHP patients [5, 27].

We found that no studies shared the exact same surgical intervention. Many have similarities, e.g. the diamond-shaped release from Ilizaliturri et al [22], which is reused in several studies. Though stating that they use a diamond-shaped release, the following studies apparently modified the surgical technique [12, 24, 68], resulting in a new intervention. Similarly, three studies [10, 39, 44] had Brignall & Stainsby inspired techniques [7]. Notably, not a single study was able to reproduce identical outcomes by using a previously described technique.

Our review has strict inclusion criteria with a focus on isolated ITB surgery and is the largest of its kind to date based on numbers of included studies, but other reviews have been performed on topics related to our review. In a review by Koulischer et al. [26], conservative and surgical management modalities of GTPS were evaluated. This review included six studies, of which four occur in our review [10, 12, 43, 57], but also Goevart et al. [17] and Baker et al. [2] who described osteotomy and isolated bursectomy with no ITB surgery, respectively. Overall, the review found comparable results to ours, with a lack of consensus regarding surgical technique, good short-term results, and only studies with level-4 evidence, emphasizing the need for larger, prospective studies.

A review on treatment of CSE by Pierce et al. [41] included seven studies, six of which are also included in our review [22, 39, 42, 44, 68, 70], with one study excluded in our review due to a pediatric population [69]. This review found the majority of studies to be small case series with short term follow-up utilizing several surgical techniques. They concluded surgery to be a safe and effective treatment of external snapping hip, but also encouraged future research to focus on larger randomized studies regarding optimal surgical technique.

A review by Reid et al. [51] included 16 studies, where three are included in this review [10, 43, 57]. The review examined general surgical management of GTPS and included studies with gluteal tears (*n* = 8). Baker et al [2] and Govaert et al [17] were excluded from our review as stated above, and also Chirputkur et al [9] who included a THA patient, Larose et al [28] with only abstract available, and Wiese et al [63] with a pediatric population. Reid et al. [51] found that ITB lengthening techniques varied between studies, that studies had poor methodological quality and were predominantly single surgeon retrospective case series. Like our review, it was emphasized that larger, prospective, long term follow-up studies with valid outcome measures are needed.

### Limitations

Our review has limitations. It became evident that no RCT or prospective observational studies have directly compared different surgical ITB interventions, nor evaluated the long-term clinical and patient related outcome for ITB surgery at the hip. All studies, except for two [3, 14], were retrospective studies (level IV evidence). This induces inherent selection- and information bias, with an additional risk of publication bias against negative findings. Furthermore, confounding by indication (“surgical bias”) could have an impact on the results obtained in this paper and provide a major concern. As an example, White et al [62] had an inclusion period of seven years and eight months, but included 11 patients. With the prevalence of cases in mind, this indicates a strong selection in the reported cases [30, 35, 47, 64].

The majority of the studies lacked information on comorbidities and patient characteristics. The studies used numerous definitions of the syndrome leading to surgery as well as varying outcome scores. Snapping was assessed differently at a study level. A couple of studies evaluate the snapping perioperatively by surgeon examination, some evaluated through questionnaires and a few merely stated snapping without defining how the information was obtained. Pain was registered with various pain scores. Dai et al [11] stated that all patients had complete pain relief, even though mean VAS-score was 2.75 (SD 0.73) postoperatively. Similar conclusions regarding VAS-score were made in other studies [12, 40].

A strong limitation of this study is the exclusion of the studies with “eligible, but not separable subgroups” (Fig. 1). This group composed of eight studies that all met the inclusion criteria apart from having the right characteristics of the population. These studies had patients who qualified for potential inclusion, but it was impossible to extract information from the given data, e.g. pediatric population in Brignall & Stainsby [7].

A total of 10 corresponding authors were contacted. Only one responded with adequate data material [24]. The author of one study had passed away [29]. Another author tried to accommodate our request but failed in retrieving the data [45]. Drummond et al [13] could not hand over the complete dataset set due to patient confidentiality but was included with the available data in this review. The eight of 49 patients who had a gluteal repair was an additional perioperative ad-on and not intended treatment [13]. The authors of the remaining six excluded studies did not respond [7, 9, 17, 45, 54, 55, 69].

### Implications of the review for practice and future research

Very little information exists on clinical and patient perceived long-term outcome following ITB surgery at the hip. Based on the available data presented in this review, only in cases of snapping can ITB surgery produce reliable resolution of snapping. Information on pain outcomes following ITB surgery at the hip is limited, diverse in reporting, and no firm conclusions can be made in relation to this. The current literature demonstrates a wide range of case definition, surgical techniques, and reporting quality. The true effectiveness of the treatment remains to be elucidated due to currently inadequate underlying evidence. Future work on ITB surgery should aim to overcome the limitations identified in this systematic review. A much higher level of evidence for future studies on ITB surgery should be prioritized.

## Supplementary Information

**Additional file 1:** **Table S1.** Data extraction for The Effect of Illiotibial Band Surgery at the Hip: A Systematic Review.

## Data Availability

All data generated or analyzed during this study are included in this published article.
